# Diffusion Breast MRI: Current Standard and Emerging Techniques

**DOI:** 10.3389/fonc.2022.844790

**Published:** 2022-07-08

**Authors:** Ashley M. Mendez, Lauren K. Fang, Claire H. Meriwether, Summer J. Batasin, Stéphane Loubrie, Ana E. Rodríguez-Soto, Rebecca A. Rakow-Penner

**Affiliations:** ^1^ Department of Radiology, University of California San Diego, La Jolla, CA, United States; ^2^ Department of Bioengineering, University of California San Diego, La Jolla, CA, United States

**Keywords:** imaging biomarker, breast cancer, diffusion tensor (DT) MRI, non-gaussian diffusion, restriction spectrum imaging, diffusion weighted (DW) breast MRI, diagnostic breast imaging, radiomics

## Abstract

The role of diffusion weighted imaging (DWI) as a biomarker has been the subject of active investigation in the field of breast radiology. By quantifying the random motion of water within a voxel of tissue, DWI provides indirect metrics that reveal cellularity and architectural features. Studies show that data obtained from DWI may provide information related to the characterization, prognosis, and treatment response of breast cancer. The incorporation of DWI in breast imaging demonstrates its potential to serve as a non-invasive tool to help guide diagnosis and treatment. In this review, current technical literature of diffusion-weighted breast imaging will be discussed, in addition to clinical applications, advanced techniques, and emerging use in the field of radiomics.

## Introduction

The history of the role of magnetic resonance imaging (MRI) in visualizing breast cancer dates back to the 1980s, when it was discovered that breast malignancies enhanced significantly compared to normal breast tissue with the use of gadolinium contrast-enhanced MRI (1–3). In the decades since then, an abundance of evidence has emerged supporting the use of dynamic contrast enhanced (DCE)-MRI in the breast, with applications ranging from high risk screening and lesion characterization, to preoperative staging and breast cancer surveillance (1). At present, DCE protocols have been accepted as the standard technique in the MRI evaluation of breast cancer by the American College of Radiology (ACR) (4). While DCE-MRI demonstrates high sensitivity in the detection of malignancy, it requires the administration of intravenous contrast, which is invasive, poses a potential risk for unknown long term gadolinium-related side effects, and is contraindicated in certain patient populations, such as pregnant women.

**Figure 1:**
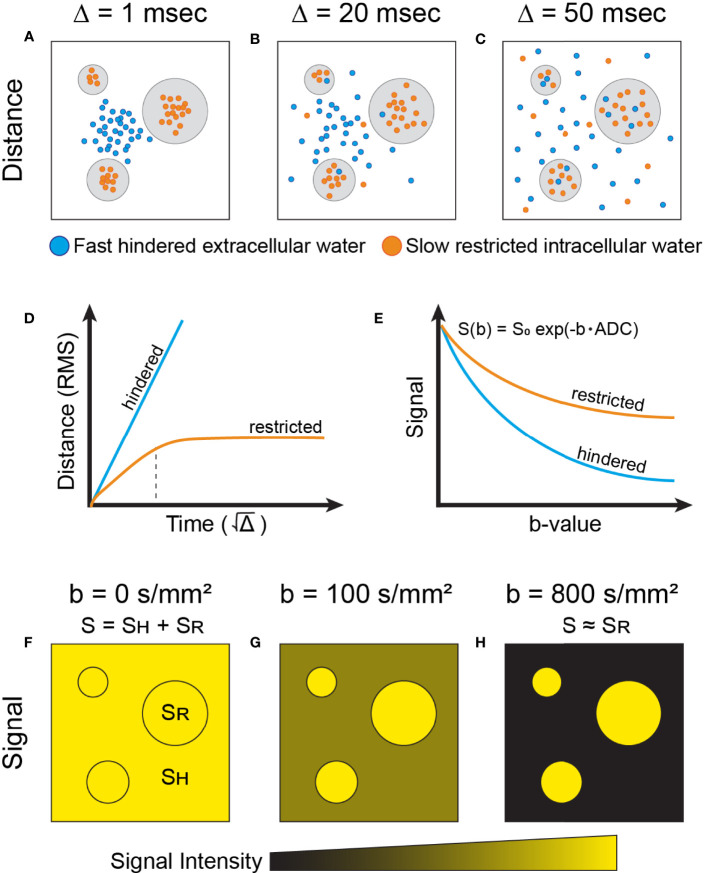
Simplified physical basis of advanced diffusion imaging. Water molecules moving at two different speeds are shown: fast-moving (free and hindered) which exist in extracellular space (blue), and slow-moving (restricted) molecules that are trapped intracellularly by the plasma membrane (orange). Note that exchange between the extra- and intracellular compartments also exists, dictated by membrane permeability. The schematic shows the dispersion of these water molecular diffusing across cellular compartments, at different timescales (Δ) of **(A)** 1msec, **(B)** 20 msec, and **(C)** 50 msec. **(D)** The root mean square (RMS) distance of water molecules experiencing hindered diffusion is linear with respect to the $\sqrt{\Delta }$ (i.e. Gaussian diffusion, blue). In contrast, slow-moving water molecules in the intracellular compartment display Gaussian diffusion behavior (linear) at very short timescales (panel **D**, orange), dictated by the compartment’s intrinsic diffusivity (5). At intermediary timescales, molecules reach the plasma membrane boundary that restricts movement, indicated by the dotted black vertical line. Past this, the net squared displacement becomes sublinear with time and is dependent on the dimensions of the compartment. To note, at very long diffusion timescales (Δ>1s), restricted water diffusion becomes principally governed by the exchange rate between the intra- and extracellular compartments (5). **(E)** In DW-MRI, the measured signal (S) decays exponentially (in the case of Gaussian diffusion) with respect to b-value due to loss of spin coherence caused by dispersion of water molecules. Thus, the signal decay from water molecules experiencing hindered diffusion (blue) is faster than from water molecules experiencing restricted diffusion (orange). The measured diffusion signal at different b-value weighting **(F–H)** reflects the relative dephasing of water molecules in different tissue compartments. At short timescales **(A, F),** the measured signal, S, contains combined information from both hindered (S_H_) and restricted (S_R_) water signal. At progressively longer timescales **(B, C, G, H)**, signal from hindered water dissipates more quickly than that from restricted water due to increased motion along the diffusion gradient axis, and the measured signal begins to arise predominantly from the restricted water signal (5). As shown in panel **(E)**, restricted water will retain more signal at higher b-values than hindered water and, correspondingly, have a lower ADC than hindered water.

Diffusion-weighted imaging (DWI) has emerged as both a complementary and potentially alternative technique to evaluate the breast. By measuring the diffusion of water molecules, quantified as the apparent diffusion coefficient (ADC), DWI provides insight into the micro-structural features of tissues (**Figure 1**). *In vivo*, the diffusion of water molecules can be categorized into three principal physical modes: free, hindered, and restricted (including partially restricted) (5–8). Free diffusion in tissues represents the random (Brownian), unhindered motion of water molecules, following a Gaussian distribution (5). Hindered diffusion represents the impeded motion of water molecules secondary to extracellular obstacles, such as high tumor cellularity (5). Restricted diffusion in tissues represents the inhibited motion of water molecules secondary to intracellular obstacles, such as a cell-membranes, and follows a non-Gaussian distribution (5). To note, whereas hindered extracellular diffusion is independent of diffusion time (dictated by the time delay between diffusion sensitizing gradients), restricted diffusion is dependent on the diffusion time, membrane permeability, and the size of the restricting cellular compartments (5).

The degree of diffusion weighting in standard DWI is measured by the b-value (s/mm^2^), a parameter determined by multiple experimental variables including the gradient strength, gradient duration, and time delay between diffusion sensitizing gradients (5, 9). The ADC value, defined as the average area occupied by a water molecule per unit time (mm^2^/s), can be estimated from the signal measured from two different acquisitions, one with diffusion weighting (non-zero b-value) and one without (b=0 s/mm^2^), according to the formula


**Figure 2:**
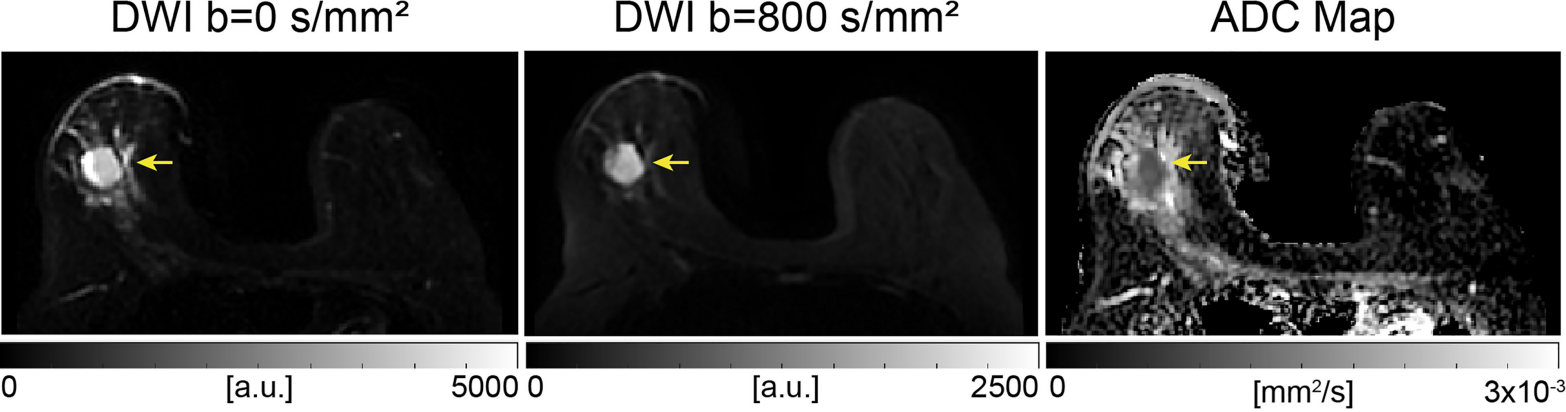
Example of conventional breast DWI at 3T, shown at b-values 0 and 800 s/mm^2^ and corresponding ADC map in a 49-year-old patient The lesion, indicated by the yellow arrow, has increased signal on b=800 s/mm^2^ images and displays lower ADC values compared to surrounding tissue, indicating a finding suspicious for malignancy. This lesion was found to be an invasive ductal carcinoma from pathology.

[1] $$S_{D}=S_{0}e^{-b\cdot ADC}$$

where S_D_ is the diffusion weighted signal intensity, S_0_ is the signal intensity without diffusion weighting and *b* is the diffusion sensitization factor in s/mm^2^ (10). Equation 1 assumes a single tissue compartment and hence mono-exponential decay (Gaussian diffusion), which is an approximation for a given tissue at a specified b-value range. At typical clinically used diffusion times (e.g. 50-100 ms), tissues with more hindered and restricted diffusion will often yield lower ADC values (3). Therefore, ADC may serve as a surrogate for tissue cellularity and thus an imaging biomarker for breast cancer (**Figure 2**). This review article will focus on standard and emerging DWI techniques and their application to breast imaging.

## Clinical Applications

### Screening

The current ACR guidelines recommend screening mammography starting at the age of 40 for women with average risk of breast cancer. For women with higher than average risk—defined as having a ≥20% lifetime risk, genetic predisposition for breast cancer, or history of radiotherapy to the chest—or a personal history of breast cancer and dense breast tissue, annual contrast-enhanced breast MRI is recommended (11). At present, DCE-MRI is the standard of care, but the role of DWI in screening is being explored.

Superior performance of DWI in the evaluation of mammographically occult and non-palpable breast cancers, particularly in women with dense breasts, compared to mammography alone has been reported (12, 13). Greater visibility of mammographically occult breast cancer on DWI compared to ultrasound was shown by Amornsiripanitch et al. (14). Compared to DCE-MRI, Pinker et al. showed that current DWI as a stand-alone tool demonstrates inferior sensitivity and diagnostic performance (15). However, the combination of DCE and DWI increased specificity and maximized diagnostic accuracy (15). Therefore, although currently not part of the BI-RADS lexicon, the inclusion of DWI in the MRI evaluation of breast cancer is encouraged by the European Society of Breast Imaging (16).

Despite evidence showing the high diagnostic accuracy of breast MRI, the financial cost and long acquisition times limit widespread implementation as a screening method in women of average risk (17). These limitations inspired the development of abbreviated breast MRI (abMRI) protocols (17). A meta-analysis of five studies found that abMRI protocols, which included first contrast-enhanced acquisition subtracted (FAST) sequences, demonstrated comparable sensitivity and specificity to standard MRI protocols in the setting of breast cancer screening (17).

Unenhanced abbreviated protocols with DWI sequences have been developed to address the drawbacks of DCE imaging, including cost, invasiveness, and safety concerns regarding the potential long-term effects of gadolinium. Studies showed comparable specificity of unenhanced abbreviated protocols that include DWI compared to either abbreviated contrast enhanced protocols or standard full DCE-MRI acquisitions (12, 13, 18–22). However, several of these studies evaluated cohorts with known malignancy (12, 13, 18), and many found that abbreviated DWI had lower sensitivity than DCE-MRI (12, 13, 18, 19, 21). Unenhanced abbreviated protocols are partly limited by decreased lesion conspicuity and lower interreader agreement (18, 19, 21, 22). Overall, results suggest that an unenhanced abbreviated protocol can maintain high diagnostic performance and represent a potential time- and cost-effective adjunct to conventional screening protocols.

### Lesion Detection and Characterization

Among the available breast imaging modalities, DCE-MRI has been established as the most sensitive in the detection of malignancy (23). Shared imaging features between benign and malignant lesions, however, limit specificity (23). The addition of DWI to DCE-MRI may offer a way of increasing diagnostic accuracy through improved specificity (24). A meta-analysis of 14 studies showed a pooled sensitivity and specificity of 91.6% and 85.5% for DCE-MRI with DWI, which was superior to DWI (86% and 75.6%) and DCE-MRI (93.2% and 71.1%) alone (25). These findings agree with other studies suggesting improved lesion characterization with multiparametric MRI (26–28). For example, a study by Pinker et al. evaluated the feasibility and diagnostic accuracy of multiparametric MRI (DCE imaging and DWI) at 7T and also found increased specificity compared to DCE-imaging alone, suggesting the addition of DWI as well as high resolution imaging may contribute to improved diagnostic accuracy (26). The added specificity from DWI holds potential to lower the false positive rate and decrease the number of unnecessary breast biopsies without missing malignancies (28).

Numerous studies have shown that DWI can be used to differentiate malignant from benign breast lesions, owing to the significantly hindered and restricted diffusion in breast cancers. A recent meta-analysis by Baxter et al. included 65 studies that evaluated the diagnostic performance of DWI and found a pooled sensitivity, specificity, and AUC of 89%, 82%, and 0.92 (29), respectively, which is comparable to results from multiple additional meta-analyses (30–32). Subgroup analysis showed that diagnostic performance was not significantly associated with the number or choice of b-values, field strength, or method of region of interest (ROI) segmentation (29).

Despite the comparable diagnostic performance of ADC across studies, threshold values varied. Small sample sizes with various proportions of lesion subtypes, differing field strengths, and selection of b-values have been suggested to contribute to this discrepancy. A recently published meta-analysis by Surov et al. aimed to provide clinically relevant information regarding use of ADC values in the differentiation of malignant and benign breast lesions (33). This analysis included 123 studies from across the world and a total of 13,847 breast lesions. The reported pooled mean ADC values for malignant versus benign breast lesions were 1.03 × 10^− 3^ mm^2^/s, 95% CI (1.01–1.05 × 10^− 3^ mm^2^/s) and 1.50 × 10^− 3^ mm^2^/s, 95% CI (1.45–1.55 × 10^− 3^ mm^2^/s), respectively (33). This study found that all benign lesions had ADC values above 1.0 × 10^− 3^ mm^2^/s, independent of field strength, choice of b-values, and ROI delineation technique (33). However, the study also demonstrated considerable overlap of malignant and benign lesions in the ADC range between 1 and 2 × 10^− 3^ mm^2^/s, which limits the clinical use of the proposed threshold value (33).

Diffusion-weighted imaging has also demonstrated potential in differentiating between invasive ductal carcinoma (IDC) and ductal carcinoma *in situ* (DCIS) (34–36). A meta-analysis of 15 studies showed a significantly higher ADC value in DCIS (0.92-1.56 × 10^− 3^ mm^2^/s) compared to IDC (0.89-1.31 × 10^− 3^ mm^2^/s) lesions, highlighting the microstructural differences between the two pathologies, potentially providing a noninvasive means of lesion characterization (34). Subgroup analysis stratified by ethnicity found lower ADC values in IDC compared to DCIS in the Asian population but not in Caucasians. Smaller sample size of Caucasian patients in this study (293 versus 858 Asians) may contribute to the differing results, as well as genetic and environmental differences (34).

### Prognostic Factors

Prognostic factors for breast cancer are used to predict survival, guide treatments, and stratify patients into clinical trials. While some of these factors, such as stage or tumor size, can be provided by imaging, several others rely on pathologic diagnosis. The use of DWI has been explored as a potential non-invasive method of predicting prognostic factors. The driving hypothesis behind these studies is that malignant lesions demonstrate high proliferation, which causes the ADC values of tissues to decrease as a result of increased cellularity (37). Tumors with increased angiogenesis are suggested to display relatively higher ADC values from increased vascular permeability and increased extracellular fluid, although this hypothesis has not yet been validated (37). Several studies have evaluated the association of ADC values and prognostic factors in breast cancer, including tumor subtype, lymph node metastases, hormone receptor expression, and histologic grade, among others.

#### Lymph Node Metastasis

The identification of lymph node metastases is necessary for accurate staging of breast cancer, which in turn affects treatment planning and prognosis (38, 39). Tissue sampling remains the gold standard but is invasive and prone to sampling error (39). As a surrogate for underlying cellularity, DWI may provide a noninvasive way of evaluating the axilla. A meta-analysis of 10 studies and 2305 lymph nodes showed a significantly lower ADC for metastatic lymph nodes (benign: 0.75-1.77 × 10^-3^ mm^2^/s vs. metastatic: 0.69-1.37 × 10^-3^ mm^2^/s), with a pooled sensitivity and specificity of 89% and 83%, respectively (39), similar to results of a few other studies (40–42). A handful of studies, however, including a large multicenter analysis, found no correlation between ADC values and lymph node involvement (43–46).

#### Hormone Receptor Expression

The correlation between ADC and hormone receptor expression has also been explored, with varied results. A meta-analysis of 6 studies showed a negative correlation between ADC values and estrogen receptor (ER) and progesterone receptor (PR) expression (47), which is consistent with the results of a few additional studies (37, 44, 48). Other groups, however, found no association with ER or PR expression (40, 45, 49, 50). A positive correlation between ADC values and human epidermal growth factor receptor 2 (HER2) expression was shown by a few groups (40, 41, 45, 48, 51), whereas others found no association (43, 44, 46, 50, 52, 53). Conflicting results were also reported regarding histologic grade, with some studies demonstrating decreased ADC values with increasing grade (41, 42, 53, 54) and others not finding a significant association (40, 52, 55, 56). Most studies found no significant association between ADC values and tumor size (42, 44, 46, 52, 53). Multiple factors may contribute to conflicting results, including differences in study design, technical parameters, and tumor types evaluated.

#### Histopathologic Subtype

The recommended treatment for breast cancer is highly dependent on biological subtype. For example, in terms of systemic treatment, Luminal A breast cancers generally only receive endocrine therapy, whereas the addition of cytotoxic therapy is indicated for most patients with Luminal B and triple negative breast cancer (57). Immunohistochemistry remains the gold standard for subtype classification but is costly and invasive. Multiple groups have investigated the potential for DWI to predict molecular subtype. A meta-analysis by Meyer et al. compared the ADC values between breast cancer subtypes and included 28 studies comprising 2990 lesions, of which 28.9% were classified as Luminal A, 30.1% Luminal B, 20% HER2 enriched, and 21% triple negative (58). Pooled data showed mean ADC values of 0.99 × 10^–3^ mm^2^/s (95% CI 0.94–1.04 × 10^–3^ mm^2^/s), 0.97 × 10^–3^ mm^2^/s (95% CI 0.89–1.05 × 10^–3^ mm^2^/s), 1.02 × 10^–3^ mm^2^/s (95% CI 0.95–1.08 × 10^–3^ mm^2^/s), and 0.99 × 10^–3^ mm^2^/s (95% CI 0.91–1.07 × 10^–3^ mm^2^/s) for these four subtypes, respectively (58). The large overlap in ADC values between subtypes is consistent with the results from a multicenter analysis by Surov et al., which found mean ADC values of 1.01 ± 0.22 × 10^–3^ mm^2^/s, 0.95 ± 0.23 × 10^–3^ mm^2^/s, 1.04 ± 0.23 × 10^–3^ mm^2^/s, and 0.95 ± 0.17 × 10^− 3^ mm^2^/s for the four subtypes, respectively, suggesting that ADC values may not be a useful predictor of molecular subtype (43).

The proliferation index, Ki-67, is a component of the subtype classification differentiating Luminal A from Luminal B breast cancer, and therefore directly affects treatment strategy. A meta-analysis by Surov et al. found a weak negative correlation (ρ=-0.22) between ADC values and Ki-67 in breast cancers (59), consistent with the findings of multiple other studies (40, 41, 43, 46, 50, 54, 55). Comparison across studies is limited, however, due to different cutoff values in the classification of high proliferation, with some using 14% and others 20%. Although statistically significant, the association is considered too weak to be clinically useful as an imaging biomarker in this context.

Histogram analysis of ADC was performed by a few groups to capture tumor heterogeneity and to determine if additional metrics were associated with prognostic factors. A study by Horvat et al. showed that the maximum ADC value based on a two-dimensional (2D) ROI on the whole tumor differentiated luminal from non-luminal cancers with an AUC of 0.685 (37). Significant overlap in ADC values between subgroups was also shown in this study, but results suggest that whole tumor segmentation may better reflect tumor heterogeneity and the different underlying architecture among molecular subtypes. Another study evaluated the added value of the entropy of ADC values, a measure of the variation in the volumetric ADC histogram and a potential surrogate for underlying microstructure heterogeneity. Results showed that the ADC entropy values differed among Luminal A, Luminal B, and triple negative phenotypes (48).

Peritumoral edema associated with breast cancer has been reported to correlate with aggressiveness and portend a poor prognosis (60–62). It has been hypothesized that neovascularity and increased vascular permeability associated with aggressive malignancies are responsible for the peritumoral edema seen on MRI (62). Therefore, evaluation of the peritumoral region may contribute additional pathophysiologic information. A study by Okuma et al. investigated whether the peritumor/tumor ADC ratio correlated with prognostic factors and indexes (49). Results showed a positive correlation between the peritumoral/tumoral ratio and size, grade, proliferation index, lymph node involvement, and lymphovascular invasion (49). While the ratio correlation of peritumor/tumor ADC with the Nottingham Prognostic Index (NPI) (0.5) and PREDICT (0.44) was stronger than that of tumoral (-0.28 and –0.25, respectively) or peritumoral (0.27 and 0.19, respectively) ADC values alone, the correlation was still considered limited to moderate. Additional studies are needed to determine if the peritumoral/tumoral ADC ratio provides any value in the prognostication of breast cancer (49).

### Predicting and Monitoring Treatment Response

Neoadjuvant chemotherapy (NAC) is commonly used in the treatment of locally advanced or large breast cancer to downstage the disease and potentially allow for breast-conserving therapy (63). The ability to non-invasively evaluate treatment response not only impacts clinical management, but also confers prognostic information, with improved outcomes seen in patients with complete pathologic response. DCE-MRI is the most commonly used modality to evaluate treatment response but is limited in the ability to differentiate residual tumor from treatment related changes, including scarring, necrosis, and reactive inflammation (64). DWI offers a potential alternative or complementary technique to overcome those limitations. The cytotoxic effects of chemotherapy disrupt cell membranes and decrease tumor cellularity, which theoretically should result in increased ADC values.

Multiple meta-analyses found that DWI could detect pathologic complete response (pCR) with a pooled sensitivity and specificity of 0.8-0.89 and 0.72-0.85, respectively (65–67). The criteria used to define complete pathologic response differed among the included studies, which partially limits comparison. The DWI metrics also varied, with some studies using the change in ADC (ΔADC) with treatment, pre-treatment ADC, post-treatment ADC, or a combination of all three to determine treatment response. Chu et al. compared the different metrics and found that the pooled specificity of the ΔADC was comparable to the post-treatment ADC, but significantly higher than that for the pre-treatment ADC group (67). This finding is partially supported by the mixed results from multiple smaller studies that investigated the ability of pre-treatment ADC to predict treatment response (68–73). While this suggests that pre-treatment ADC values may not represent as reliable a predictor of pCR compared to the ΔADC and post-treatment ADC, multi-center trials with larger population sizes and standardized acquisition protocols would be needed to make this determination and validate the use of ADC for this clinical use.

The results from the American College of Radiology Imaging Network (ACRIN) 6698 trial further demonstrate the ability of DWI to predict pathologic response (74). In this clinical trial, 272 women with breast cancer underwent DW-MRI prior to NAC, 3 weeks into treatment, 12 weeks into treatment, and after completion of chemotherapy. The percent change in tumor ADC from baseline was measured at each time point. Results showed that the ΔADC was somewhat predictive of pCR at mid-treatment (12 weeks) (AUC 0.6; 95% CI: 0.52-0.68; P= 0.017) and after treatment (AUC 0.61; 95% CI: 0.52-0.69; P = 0.013). Significantly increased treatment related ΔADC values in patients with pCR supports the findings from multiple single center studies (68, 69, 72, 75–77).

A meta-analysis by Gu et al. evaluated the role of MRI in the detection of pCR after neoadjuvant treatment in patients with breast cancer and found that DCE-MRI demonstrated superior pooled specificity in terms of identifying residual tumor (0.92 versus 0.85) while DWI maintained higher sensitivity (0.93 versus 0.64) (65). The relatively low sensitivity of DCE-MRI may be secondary to nonspecific contrast enhancement from post-treatment changes, including reactive inflammation, necrosis, and perilesional edema, or from co-existing DCIS (65). The diagnostic accuracy of DCE-MRI was greater than ultrasonography and mammography (0.96 versus 0.66 and 0.53) but not significantly different than PET/CT (0.99), which demonstrated higher sensitivity of 0.9 (65). Results suggest that DCE-MRI combined with DWI or PET/CT in these patients may improve predictive accuracy (65).

## Validation and Technical Considerations

Technical validation is necessary prior to translation of quantitative imaging biomarkers into community practice. This process involves standardization of acquisition protocols and demonstration of acceptable repeatability and reproducibility to ensure consistent results across practice settings.

### Repeatability and Reproducibility

For implementation in clinical practice, a quantitative imaging biomarker should demonstrate high accuracy and precision, reflected in repeatability and reproducibility. Repeatability represents the precision of repeated measures taken under identical conditions in a short amount of time, while reproducibility represents the precision of repeated measures wherein some aspect of the procedure is changed (e.g. different field-strength scanners) (78). Understanding the factors which affect repeatability and reproducibility, such as image acquisition parameters and data analysis, is necessary for the development of a useful imaging biomarker.

Multiple small single center studies have shown good repeatability and reproducibility of ADC measurements in normal (79–82) and malignant breast tissue (80, 81, 83). The ACRIN 6698 trial evaluated the repeatability and reproducibility of ADC measurements in a multi-institution, multi-MRI platform clinical setting (84). Results demonstrated excellent repeatability [within-subject coefficient of variation = 4.8% (95% CI 4.0-5.7%)] and reproducibility [interreader intraclass correlation coefficient (ICC) = 0.92 (95% CI 0.80-0.97) and intrareader ICC = 0.91 (95% CI 0.78-0.96)] independent of field strength when using a standardized DWI protocol and quality assurance (QA) procedures (84). This study represents an important step in the validation of ADC as a quantitative imaging biomarker by showing high precision in a multi-institution setting.

The Quantitative Imaging Biomarkers Alliance (QIBA) previously excluded breast from the QIBA Profile for DWI in 2017 due to a lack of reproducibility data in the literature. In light of the increasing evidence, the QIBA added breast to the DWI Profile in 2019, providing guidance on protocol design (**Table 1**), quality assessment, and image analysis, with additional details provided in the following sections (85).

**Table 1:** Protocol guidance for diffusion weighted imaging of the breast provided by the QIBA.

Field Strength	1.5 or 3 T
Acquisition sequence	Diffusion-weighted Single-Shot Echo Planar Imaging (ss-EPI)
Receive Coil type	Ideal/Target: 5-16 channel bilateral breast coil Acceptable: 4 channel bilateral breast coil
Fat Suppression	On
Number of b-values	Ideal: ≥ 4 Target/Acceptable: 3 (including one b=0-50, one 100, and one at highest b-value Acceptable: 2 (including one b=0-50 s/mm^2^ and one at highest b-value)
Minimum highest b-value strength	Target/Ideal: b=600-800 s/mm^2^ Acceptable: 600 s/mm^2^
Diffusion directions	Target/Ideal: 3-orthogonal, combined gradient channels Acceptable: 3-orthogonal, single gradient channels
Slice Thickness	Ideal: 4 mm Acceptable: 5 mm
Gap thickness	Ideal: 0 mm Acceptable:1 mm
Field-of-view	Ideal/Target/Acceptable: 260-360 mm (complete bilateral coverage)
Acquisition matrix	Target/Ideal (128-192) x (128-192), or 2.8- 1.8 mm in-plane Acceptable: 128 x 128, or 2.8 mm in-plane resolution
Plane orientation	Transversal-axial
Half-scan factor	Acceptable/Target: >0.65
Phase-encode/frequency-encode direction	Anterior-Posterior/Right-Left or Right-Left/Anterior-Posterior
Number of averages	Ideal/Target: 3-5 Acceptable:2
Parallel imaging factor	Ideal: ≥ 2 Target/Acceptable: 2-3/2
TR	Ideal/Target/Acceptable ≥ 4000 ms
TE	Ideal/Target: minimum TE (50-100ms) Acceptable: < 114 ms
Receiver Bandwidth	Ideal/Target: maximum possible in frequency encoding direction (minimum echo spacing) Acceptable: > 1000 Hz/voxel

Definitions provided by the QIBA:

ACCEPTABLE: Actors that shall meet this specification to conform to this profile.

TARGET: Meeting this specification is achievable with reasonable effort and adequate equipment and is expected to provide better results than meeting the ACCEPTABLE specification.

IDEAL: Meeting this specification may require extra effort or non-standard hardware or software, but is expected to provide better results than meeting the TARGET.

The European Society of Breast Radiology (EUSOBI), which works closely with the QIBA, created an international breast DWI working group consisting of MRI physicists, clinical breast MRI experts, and MRI vendor representatives from 16 countries (16). The group published the first consensus and mission statement in 2020, proposing acquisition parameters for DW sequences and ROI segmentation recommendations for clinical application with the goal of improving protocol standardization across institutions and attaining standardized ADC values. The group’s future efforts will focus on addressing factors that alter precision and the development of quality control, with a goal of progressing towards widespread implementation of quantitative breast DWI (16).

### Acquisition Techniques

The QIBA DWI profile currently recommends utilizing a single-shot echo planar imaging (ss-EPI) acquisition sequence for diffusion weighted breast imaging (85). In ss-EPI, the imaging data from all k-space is obtained with a single radio-frequency excitation, allowing for shorter acquisition time and decreased motion artifact (6, 86). However, ss-EPI is strongly affected by susceptibility artifacts and typically has low spatial resolution. These limitations can be mitigated by adequate fat suppression, use of parallel imaging, and shimming (6, 86).

Alternative acquisition techniques have emerged to address these limitations and have demonstrated potential for improved image quality in DWI breast imaging. In general, these techniques reduce the readout duration, thus shortening the time during which the signal is affected by field inhomogeneities that cause distortion artifacts.

**Readout-segmented echo planar imaging (rs-EPI)** is a multi-shot technique that divides k-space into multiple segments, allowing for decreased echo spacing, reduced geometric distortion, and improved resolution (87). Multiple studies have demonstrated superior breast lesion conspicuity and image quality with rs-EPI compared to ss-EPI (88–91). Inter-reader agreement of known mass and non-mass lesions was evaluated in two studies: DCE-MRI and rs-EPI collected with b-values of 0 and 850 s/mm^2^ resulted in comparable morphologic lesion assessment and diagnostic performance (21, 92). These findings suggest rs-EPI as a potential alternative to DCE-MRI. However, improved image quality with rs-EPI is often at the expense of increased acquisition times, and lesion conspicuity remains inferior to DCE-MRI

**Figure 3:**
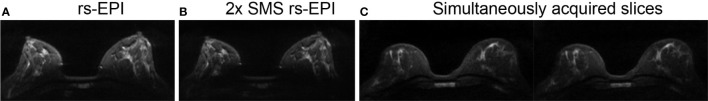
Example of SMS rs-EPI acquisition at b=800 s/mm^2^ in a 35-year-old healthy volunteer, wherein **(B)** two-fold (2×) SMS rs-EPI maintains comparable image quality as **(A)** conventional rs-EPI while reducing scan time in a 3T scanner. Panel **(C)** shows two simultaneously acquired slices used to generate a single-band equivalent image for the same patient at a different slice location (94). (Courtesy of Lukas Filli, MD, Zurich, Switzerland).

**Simultaneous multi-slice (SMS) rs-EPI** was introduced to address the increased acquisition times required with rs-EPI. In SMS imaging, multiple slices are acquired simultaneously so that the number of excitations required for the same slice coverage is reduced (93). The spatial sensitivity of multichannel array coils is subsequently used to separate the slices acquired in parallel (93). Filli et al. first demonstrated the feasibility of SMS rs-EPI in 8 healthy volunteers, comparing conventional rs-EPI to two-fold and three-fold slice-accelerated rs-EPI (**Figure 3**) (94). They found that while scan time was significantly reduced and SNR was improved with additional acceleration, ghosting artifacts and shading in the prepectoral region were more distinct (94). A more recent study by Song et al. compared image quality, lesion conspicuity, and scan time between rs-EPI and SMS rs-EPI sequences in 134 women with invasive breast cancer (95). The study found a 44% reduction in scan times, improved image quality, and enhanced lesion conspicuity with SMS rs-EPI, similar to the results of a study by McKay et al. (95, 96). Compared to conventional rs-EPI, SMS rs-EPI produced comparable AUC and ADC values in multiple studies, suggestive a potential method of reducing scan time while preserving diagnostic accuracy (94, 95, 97, 98).

**Figure 4:**
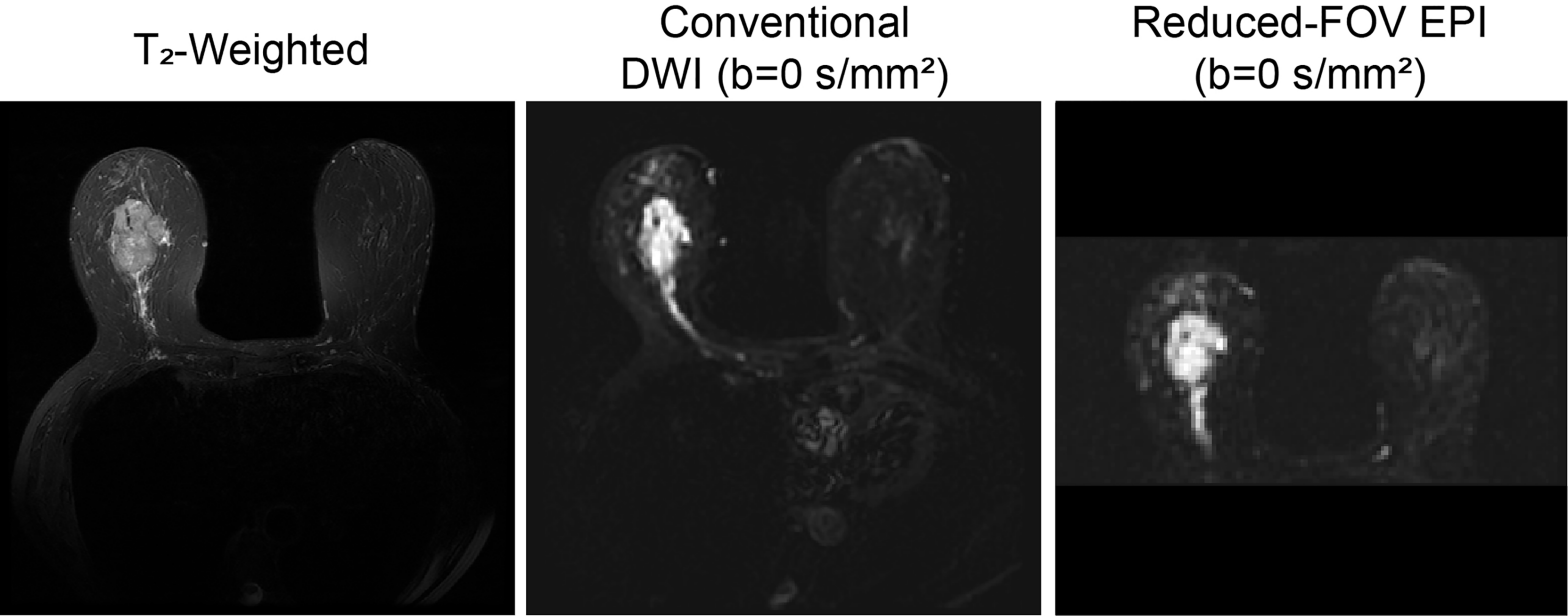
Reduced FOV EPI in a 63-year-old patient with invasive ductal carcinoma. T_2_-weighted, conventional DWI (b=0 s/mm^2^), full FOV EPI (b=0 s/mm^2^), and reduced FOV EPI (50% phase field of view) (b=0 s/mm^2^ acquired at 3T) images are shown. Reduction of percent phase encoding direction to 50% reduces geometric distortions caused by B_0_-inhomogeneity, especially in the nipple region (100).

**Reduced field of view (rFOV)** improves spatial resolution and decreases artifacts by limiting the field of view and number of k-space lines in the phase-encoding direction (99, 100). Improved image quality with rFOV compared to standard DWI techniques has been shown to enhance lesion conspicuity and morphologic assessment in the breast (101–104). Significant differences in ADC values with rFOV compared to full FOV DWI, however, may limit the utility of proposed ADC cutoff values when employing rFOV techniques (**Figure 4**) (101, 102, 104, 105).

rFOV has been used in conjunction with other acquisition strategies to further improve image quality and reduce scan time. For instance, Taviani et al. developed a single-shot image-segmented technique that combines rFOV, 2D in-plane multiband radiofrequency pulses, and a generalized parallel imaging reconstruction method to generate images with high resolution and anatomical fidelity (106).

**Figure 5:**
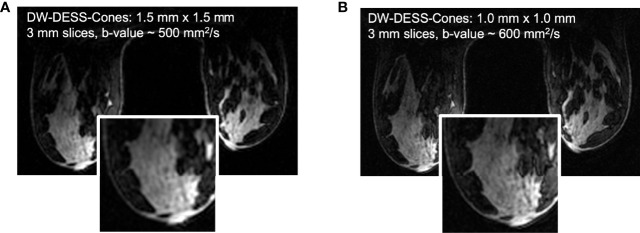
Based on the results of the initial DW-DESS-Cones investigation in the breast at 3T **(A)**, the diffusion-weighting and resolution of the method can be further increased **(B)** to better match contrast and resolution expectations for breast MRI (109). (Courtesy of Catherine Moran, PhD, Department of Radiology, Stanford University, Stanford, California, USA).

**Diffusion weighted double-echo steady state (DW-DESS)** imaging is an emerging technique that allows for rapid acquisition of high-resolution images by utilizing a short repetition time (TR) (107–110). The diffusion weighted DESS sequence acquires two echoes per radiofrequency pulse, during which a steady state of longitudinal and transverse magnetization is achieved. Multiple parameters affect the diffusion weighting in DW-DESS, such as the TE, TR, flip angle, spoiler gradient duration, and tissue relaxation and diffusion properties (107, 108, 110). A few studies have evaluated the use of DW-DESS imaging in the breast and found superior image quality and improved morphologic assessment when compared to conventional EPI DWI (108, 111). Benefits of this technique include rapid acquisition times and avoidance of EPI-associated distortions and blurring (107, 108). The DW-DESS sequence, however, is susceptible to motion artifacts, particularly with increased diffusion weighting (109). Moran et al. developed a DW-DESS-Cones method using a three-dimensional cones (non-cartesian) trajectory to address this limitation, and demonstrated significantly reduced motion artifacts (**Figure 5**) (109). At present, DW-DESS techniques do not provide a reliable quantitative measure of diffusion equivalent to ADC values, and will likely be the focus of future investigations (108, 109).

### b-Value Selection

ADC values are typically displayed as a parametric ADC map. Regions of high cell density and hence highly hindered and restricted (including partially restricted) diffusion appear hypointense on the ADC map and hyperintense on high b-value diffusion weighted images.

According to the monoexponential mathematical model (Eqn. 1), b-value selection directly affects the ADC value, signal-to-noise ratio (SNR), and contrast-to-noise ratio (CNR). With increasing b-value, ADC values theoretically decrease due to the predominance of non-Gaussian diffusion. Additionally, increased CNR with increasing b-values may improve lesion detection at the expense of decreased SNR (10). Studies aiming to identify optimal b-value selection in DWI of breast demonstrate varied results (112–115). The QIBA requires a minimum of two b-values, b=0-50 s/mm^2^ and b≥ 600 s/mm^2^, but recommends ideally acquiring 4 or more b-values, including b=0-50 s/mm^2^ (78). As more evidence emerges, particularly with advanced modeling techniques requiring multiple b-values, recommendations may become increasingly specific.

### ROI Delineation

Typically, ADC values are extracted by placing a region of interest (ROI) on the restricting lesion. The most commonly employed methods of ROI placement are whole lesion segmentation and focused segmentation, where the ROI is applied to the most restricting portion of the lesion (highest signal on DWI corresponding to lowest ADC value on ADC maps) (116–119). ROI placement has been shown to significantly affect ADC measurements, limiting the use of proposed ADC cutoff values (116–119). Compared to whole lesion segmentation, focused ROI placement demonstrates superior diagnostic accuracy in the evaluation of breast lesions in a few studies, likely on the basis of emphasizing the most restricting and thereby most suspicious portion of the tumor (117, 119). Focused segmentation allows the exclusion of region of necrosis, non-enhancement, and artifacts, resulting in an ADC value that may better represent the underlying microstructure (16, 118). Additionally, semiautomated ROI delineation algorithms, such as that developed by Rahbar et al., can improve inter-reader reproducibility of ADC measures (120). While the QIBA has not provided ROI placement standards, the EUSOBI presently recommends using a focused segmentation method—while taking care to avoid regions of necrosis, non-enhancement, and artifacts—with the goal of improving consistency of DWI across institutions (16).

## Advanced and Emerging Techniques

To address the shortcomings of the monoexponential ADC model in capturing the complex tissue micro-structure in the breast, several advanced diffusion models have been developed and will be explored in this section.

### Diffusion Tensor Imaging

Diffusion tensor imaging (DTI) is a quantitative technique within DWI that measures the diffusion directionality (anisotropy) of water molecules by applying at least 6 directional diffusion gradients, providing a three-dimensional representation of diffusion (121–125). The diffusion tensor model is mathematically represented by a symmetric matrix of six parameters: three orthogonal eigenvectors (ν_1_, ν_2_, ν_3_), reflecting the direction of diffusion, and three corresponding eigenvalues (λ_1_, λ_2_, λ_3_), reflecting the degree of diffusion in each orthogonal direction (121–125). From the eigenvalues, DTI metrics are derived (121, 125). The most common DTI metrics studied are fractional anisotropy (FA), or the fraction of diffusion that is anisotropic on a scale from 0 to 1, and mean diffusivity (MD), or the average of tensor’s eigenvalues, also represented as the ADC (121, 125). Additional DTI parameters include maximal anisotropy (MA), relative anisotropy (RA), volume ratio, geodesic anisotropy, and radial diffusion. Maximal anisotropy represents the difference between the highest and lowest value of anisotropic water movement (λ_1_ - λ_3_) (126). Relative anisotropy is the ratio of the standard deviation to the mean of the three eigenvalues, ranging from 0 to √2, with 0 representing isotropic diffusion and the √2 representing diffusion in a single direction (126). The volume ratio is the ratio of the ellipsoid to spherical, ranging from 0 to 1, with 1 reflecting isotropic diffusion (127). Radial diffusivity is the average of the two smaller eigenvalues (λ_2_ and λ_3_) (128)

Normal breast architecture is comprised of multiple lobules with a complex ductal network with surrounding fibrous stroma and intervening fatty tissue. Within small ducts, it has been suggested that the diffusion of water molecules is anisotropic and DTI values may provide information regarding pathophysiologic changes in tissue microstructure (123, 129). A few studies have evaluated DTI parameters in women with normal breasts and found significant regional differences, with increased FA within the periphery and posterior aspects of the breast compared to the central breast, which is postulated to reflect anisotropic diffusion within smaller, collapsed ducts peripherally and posteriorly (123, 129, 130). A study by Plaza et al. showed no association between DTI parameters and fibroglandular tissue composition, but found a significantly lower λ_1_ in normal breasts with moderate/marked background parenchymal enhancement (BPE) compared to those with minimal/mild BPE (131). Other studies have observed that DTI parameters are resistant to physiologic differences in breast tissue composition due to their unique ability to track underlying ductal microstructure (123, 132, 133). In comparison to DCE, certain DTI parameters have also shown superior tumor conspicuity in lactating patients with pregnancy-associated breast cancer (134). Background parenchymal enhancement is a challenge among this patient population. In a study by Nissan et al., CNR for lactating patients with pronounced BPE were higher on λ_1_, λ_2_, λ_3_, and MD (1.81 ± 0.67, 1.95 ± 0.87, 1.79 ± 0.83, respectively) maps as compared with those of DCE images (0.82 ± 0.49) (p < 0.005, for all) (134). These correspond to an increase in CNR of up to 138% by DTI-derived parameters, compared to DCE. DTI parameters, much like ADC (132, 135–137) have been shown to be resistant to changes in the breast parenchyma (131, 132, 134), unlike DCE (138, 139), which further demonstrates the utility of diffusion MRI as an effective adjunct to DCE.

Disruption of the breast architecture has been suggested to alter anisotropic indices, and which may therefore serve as potential imaging biomarkers of malignancy. A comprehensive meta-analysis by Wang et al. evaluated the diagnostic performance of DTI metrics in discriminating benign versus malignant breast lesions (140). This analysis included 16 studies with a total of 1636 patients and found significantly higher FA (0.15-0.55 versus 0.02-0.13), and lower MD (0.71-1.62 versus 1.08-1.91), λ_1_ (0.97-1.62 versus 1.19-2.15), λ_2_ (0.95-1.29 versus 1.50-1.68), and λ_3_ (0.78-1.12 versus 1.20-1.56) in malignant lesions compared to benign lesions (140). Decreased diffusion coefficients may be in part secondary to increased cellularity within the malignancy, as well as ductal involvement of neoplastic cells (140). Pooled FA was increased in malignant lesions, but individual studies showed conflicting results (140). For example, Furman-Haran et al. found no difference in FA between malignant lesions and contralateral breast parenchyma, but did find that the absolute maximal anisotropy index (λ_1_-λ_3_) differentiated the tissues (lesion: 0.51 x 10−3, mm2/sec, versus normal: 0.84 x 10^−3^ mm^2^/s, p<0.001) (126). Increased FA values in malignancy are postulated to reflect disorganized architecture with regional necrosis or hemorrhage, that results in increased diffusion along certain directions but hindered diffusion in others (126, 140). If regions of necrosis or hemorrhage are large enough, however, diffusion of water molecules may be uninhibited and result in reduced anisotropy, which may explain why some of the included studies concluded that FA could not distinguish malignant from benign lesions (140). Furthermore, normalized anisotropic indices such as FA are subject to the inherent mean diffusivity of the underlying tissue, which may differ by lesion subtype (126, 140). Subgroup analysis revealed a significantly lower MD value among invasive breast cancer lesions compared to DCIS (140). Overall, λ_1_ demonstrated the highest diagnostic accuracy, with a pooled sensitivity of 93%, specificity of 92% and AUC of 97%. These findings suggest MD and λ_1_ may be clinically useful markers of malignancy (123, 128, 140, 141).

An additional meta-analysis performed by Baxter et al. compared the diagnostic performance of DWI, DTI, and intravoxel incoherent motion (IVIM) in the characterization of breast lesions (29). In this analysis, λ_1_ also demonstrated the highest diagnostic accuracy among DTI metrics, with a pooled sensitivity of 93%, specificity of 90% and AUC of 94% (29, 123, 128, 141). Overall, the diagnostic performance of DWI, DTI and IVIM was comparable but the conclusions were limited by the low number of included studies and thereby low statistical power (29).

The association of DTI parameters with prognostic factors has been investigated by a few studies with promising results (128, 142–144). Significantly low MD and FA values were found to correlate with larger tumor size (>2 cm), high histologic grade, and axillary nodal metastases/lymphovascular invasion (142–144). Other DTI parameters were also found to be significantly associated with ER, PR, CERB-B2, Ki-67 and intrinsic subtypes (128, 143).

A retrospective study by Furman-Haran et al. included 20 women undergoing NAC and compared DTI parameters with DCE-MRI in the ability to monitor treatment response (145). Results showed that the post NAC change in multiple DTI parameters, including MD, λ_1_, λ_2_, and maximal anisotropy (λ_1_-λ_3_) differentiated responders from non-responders after NAC, with the highest AUC seen with MD, λ_1_ and λ_2_ (145). The change in FA was not statistically significant (145). Pre-NAC DTI parameters however showed low diagnostic performance in the ability to predict NAC response (145). Tumor size changes following NAC measured by DTI were of comparable accuracy to that of DCE and found to also be a significant discriminator between responders and non-responders (145). Residual tumor diameter correlated well with the postoperative pathological tumor diameter (145).

At present, no standard DTI protocol exists, with varied selection of b-values and numbers of diffusion gradients seen across studies, which may affect the resultant DTI metrics. It has also been demonstrated that DTI is prone to artifacts at high b-values and high resolution, common to other EPI-based sequences, which affect interpretation of the DTI parameters (146). A study by Yamaguchi et al. found superior diagnostic performance of DTI based on rs-EPI compared to DWI based on ss-EPI, which was attributed to improved lesion conspicuity and diminished blurring artifact (144). Further studies are needed to establish a standardized protocol and threshold values for practical clinical use.

### Intravoxel Incoherent Motion (IVIM)

Diffusion-weighted imaging and subsequent ADC measurement are influenced by both Gaussian and non-Gaussian diffusivity, which includes microcapillary perfusion. The intravoxel incoherent motion (IVIM) model, first introduced in 1986 by Le Bihan et al., provides a method to separate the contribution of micro-perfusion from tissue diffusivity to the diffusion-weighted signal (147). Using the following biexponential decay model,


[2] SS0=fe−b(D+D∗)+(1−f)e−bD

and multiple b-values, the following parameters can be attained: water diffusion through tissue (D or D_t_), pseudo-diffusion from perfusion (D^*^, D_p_ or D_f_) and the perfusion fraction (f, f_p_, or f_IVIM_). First applied to the breast in 2011 by Sigmund et al., the IVIM model has been increasingly studied and shown promise in the evaluation of breast lesions (148).

**Figure 6:**
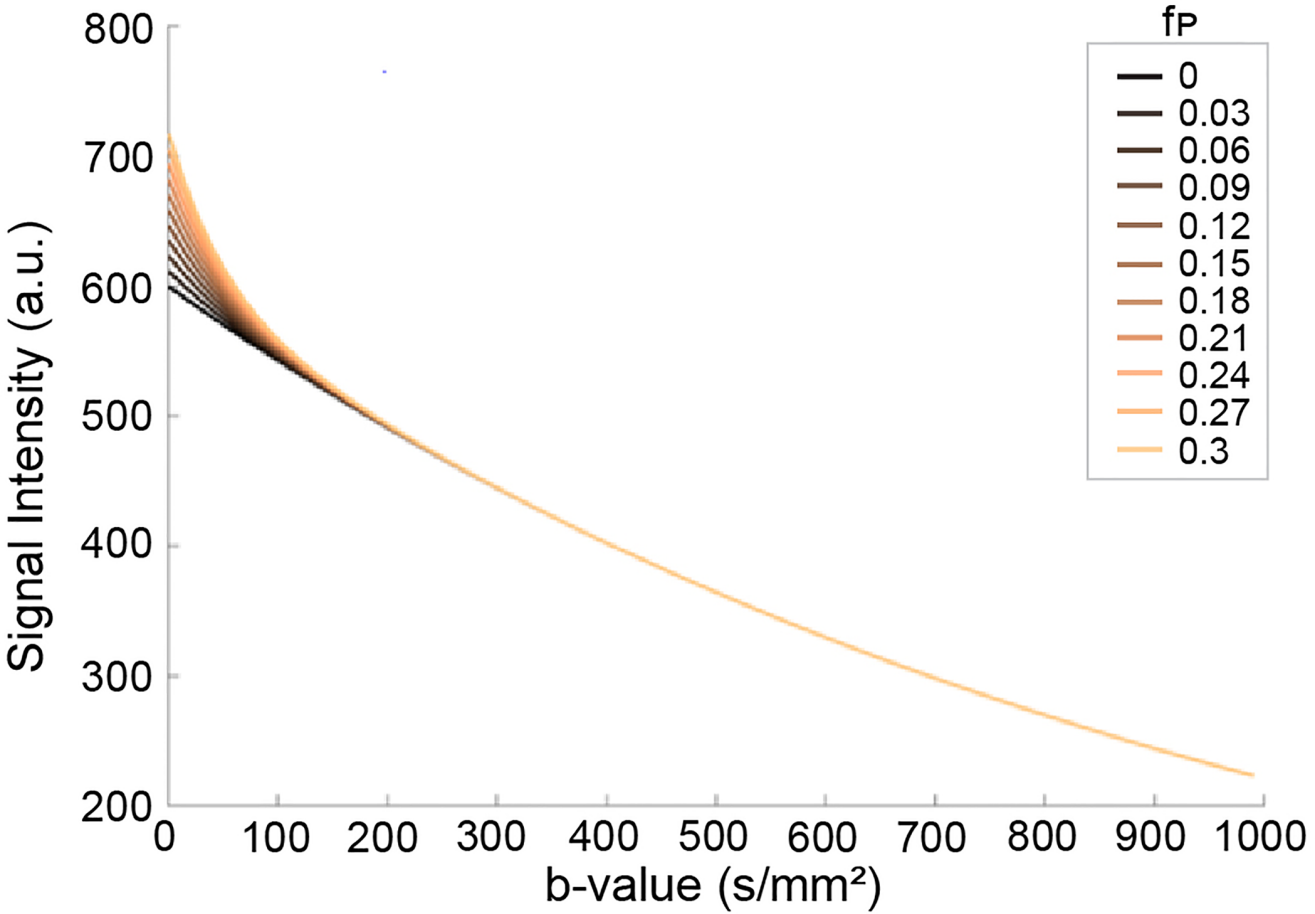
Effects of pseudo-diffusion on DWI signal. Signal curves in the presence of increasing IVIM effects deviate from the simple mono-exponential curve (f_p_=0, black line). The components have unique coefficients D_t_ = 0.001 s/mm^2^ and D* = 0.02 s/mm^2^ with relative proportions given by the pseudo-diffusion fraction f_p_ (164). (Courtesy of Igor Vidić, PhD, previously at the Department of Physics, Norwegian University of Science and Technology, Trondheim, Norway).

The IVIM parameters have been shown to aid in the discrimination of malignant from benign breast lesions. In multiple studies, malignant lesions showed significantly decreased tissue diffusivity (D_t_) values and increased perfusion fraction (f_p_) values compared to benign lesions and normal breast parenchyma (36, 149–162). A recently published review of fifteen studies yielded sensitivity of 87 ± 10% and specificity of 79 ± 17% for D_t_ in malignant lesions, and a sensitivity of 81 ± 7% and specificity of 75 ± 3% for f_p_ (163). In terms of diagnostic performance, multiple studies found that at least one IVIM metric, most consistently D_t_, outperforms ADC, with one study finding an increased AUC when D_t_ and f_p_ are combined (0.84 vs 0.75 for D_t_ alone, and 0.79 for f_p_ alone) (**Figure 6**) (36, 152–155, 157–159).

Direct comparison and correlation of IVIM parameters with standard DCE-MRI has been performed (155, 157, 165). In a few studies, D_t_ outperformed DCE-MRI derived parameters with an overall increased AUC when IVIM and DCE-MRI parameters were combined (AUC 0.99 with combination of D_t_ and time-signal intensity curve (157); AUC 0.93 with multivariate combination of IVIM and DCE parameters (155, 157, 165). Multiparametric approaches combining IVIM and other non-Gaussian DWI parameters also have shown increased diagnostic accuracy, with one study by Lima et al. demonstrating BI-RADS equivalent scores (150). These findings suggest that the addition of IVIM metrics to standard DCE-MRI may improve diagnostic accuracy, and that IVIM may represent a non-invasive alternative to DCE-MRI.

The role of IVIM in the non-invasive identification of prognostic factors in breast cancer has also been investigated. Multiple studies found a correlation between D_t_ and ER expression (36, 56, 161, 166). Zhao et al. also found that the D^*^ and D_t_ significantly correlated with ER and PR expression and Luminal A subtypes (161). Luminal B subtypes in this study showed significantly decreased f_p_, with significantly diminished peritumoral f_p_ values among HER2 positive lesions compared to HER2 negative lesions, a finding which may reflect diminished central perfusion secondary to intratumoral necrosis (161). The IVIM parameters D^*^, f_p_ and D_t_ correlated with TNBC status, with increased f_p_ values along the tumor edge compared to other subtypes and increased peritumoral D^*^ values, which may suggest a high degree of invasiveness (161). The work by Zhao et al. showed that applying IVIM metrics to the peritumoral and tumor edge may shed light on the underlying pathophysiology.

Multiple studies found a correlation between D_t_ values and Ki-67 expression (149, 161, 162, 167, 168), with two of these studies demonstrating a correlation with f_p_ values (161, 168). Evaluation of the association of IVIM metrics with lymph node metastases and histologic grade, however, have yielded conflicting results (56, 159, 161, 166, 168).

A study by Lee et al. investigated the association of IVIM parameters with two markers of tumor angiogenesis, microvascular density (MVD) and vascular endothelial growth factor (VEGF), in patients with breast cancer using 4 different curve fitting algorithms (169). The authors found significant associations between multiple perfusion related parameters and VEGF using a linear regression model to determine D_t_ and f_p_ at high b values, and linear regression to determine D* at low b values (≤50 s/mm^2^) (169). However, no association was found between MVD and IVIM parameters obtained by the 4 different curve fitting algorithms, and additional studies are needed to determine if there is a correlation (169).

Histogram analysis of IVIM parameters performed by a few groups demonstrated the potential to distinguish breast cancer subtypes and additional prognostic factors (36, 166, 170). As opposed to the majority of studies where the average values for IVIM metrics are obtained, histogram analysis appears to provide additional information of the distribution of the metrics, including skewness and kurtosis, which better reflect tumor heterogeneity.

A few studies evaluating the ability of IVIM parameters to predict treatment response have shown conflicting results. Two studies reported increased D_t_ values following NAC in the responder (or pCR) group (171, 172), whereas two other studies did not find significant differences between groups (70, 173). The small sample sizes in these studies may account for the observed differences, warranting further investigation with larger cohorts.

Direct comparison across studies is limited due to the variability in the methods of image acquisition and data analysis, as the choice of curve fitting methods and b-values have been shown to affect IVIM metrics (174, 175).

The b-value selection significantly affects IVIM metrics. A threshold value of 200 s/mm^2^ has been used, with perfusion effects predominating below 200 s/mm^2^ and diffusion effects predominating above 200 s/mm^2^ (150, 151, 153, 174). However, a variety of threshold b-values have been used in breast studies and there is currently no consensus on the optimal threshold or b-values choice. A study by Chen et al. aimed to determine the optimal threshold b-value and found an optimal cutoff value of 300 s/mm^2^ discriminated diffusion from perfusion effects (176). Ongoing research efforts aim to determine the optimal b-values. For example, Cho et al. compared a free (conventional constrained least squares fit) versus a segmented (two step constrained analysis) fitting method for both conventional or optimized b-values (174). This group found that the IVIM values differed significantly according to the sampling method, with a segmented method for optimized b-values showing the highest accuracy and precision (174).

Several studies have investigated different fitting and analysis methods for IVIM in order to increase accuracy and differentiation between lesion type. Suo et al. compared three frequently used calculation methods in women with biopsy proven IDC and found significantly higher precision when using either of the applied two step calculation methods compared to the conventional free fitting model (175). Most IVIM metrics differed significantly according to the calculation method, with a significantly larger f_p_ value with the free fitting model (175).

Bayesian fitting approaches have been investigated as an alternative to nonlinear least squares fitting (177, 178). The Bayesian model uses prior knowledge or assumptions of the system to provide estimates of IVIM parameters for pixels with a high degree of data fitting uncertainty, decreasing heterogeneity in the parameter maps (177). A study by While et al. compared the performance of multiple Bayesian modeling approaches with least squares-based approaches on simulated breast and liver tissue (177). In terms of relative error and estimator deviation, Bayesian approaches outperformed both full and segmented least squares-based methods (177). However, in areas of high parameter uncertainty, certain features disappeared, potentially masking important tissue characteristics and limiting interpretation (177). This study also showed that segmented least squares approach was superior to the full nonlinear approach in the breast (177).

Alternative methods of data analysis have been proposed. In one such method called the exhaustive approach, the parameters are derived from comparing the raw signal to an exhaustive database of simulated signals, comprised of a large set of parameter combinations (153). This method may provide a better estimation of IVIM metrics by eliminating the local minima issue seen in fitting models, but it requires high processing power (153). An additional method, termed the simplified approach, uses only three b-values to calculate the relative enhanced diffusivity (RED), a metric that pools the effects of ADC mapping and IVIM modeling (179, 180). A study by Teruel et al. found that the RED differentiated malignant from benign breast lesions with an overall accuracy of 90% using b-values of 0, 200 and 700 s/mm^2^ (180).

### Diffusion Kurtosis Imaging (DKI)

**Figure 7:**
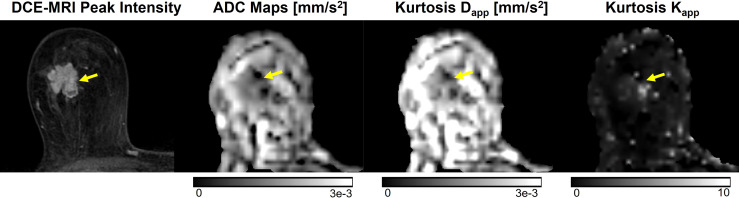
Example of DKI analysis using b-values of 0, 500, 1000, and 2000 s/mm^2^, compared to conventional ADC images using b-values of 0 and 1000 s/mm^2^ and DCE-MRI peak intensity subtraction (1 min 30 s post-contrast). Invasive ductal carcinoma in a 67-year-old patient is indicated by the yellow arrow. The lesion displays higher mean kurtosis (K_app_) than surrounding healthy tissue. Images were acquired using a wide-bore 3T scanner, and mean kurtosis and diffusivity (D_app_) were calculated as previously demonstrated (181).

Diffusion kurtosis imaging (DKI) is an extension of DWI in which both Gaussian and non-Gaussian diffusion distributions are quantified, providing added insight into the tissue microstructure (181). DKI yields the parameters mean diffusivity (D), representing Gaussian diffusion, and mean kurtosis (MK, K), a unitless metric representing the degree of non-Gaussian diffusion. The DKI model is the following:


[3] $$\ln\frac{S(b)}{S(0)}\approx -bD+\frac{1}{6}b^{2}D^{2}K$$

where S(b) is the DW signal with non-zero diffusion weighting, S(0) the signal without diffusion weighting, and b the diffusion weighting factor (181). As malignant lesions proliferate, increased cellularity results in decreased extracellular space and increased microstructural complexity (i.e. cell membranes and organelles), impairing Gaussian diffusion (182). The degree of deviation from Gaussian diffusion can be quantified by K, with increasing K value reflecting increasing deviation (**Figure 7**) (181).

The potential of DKI parameters in the characterization of breast lesions has been investigated. Multiple studies have found that malignant lesions demonstrate a significantly higher K (0.61-1.13) and lower D (1.01-1.52 × 10^-3^ mm^2^/s) values compared to benign lesions (K of 0.37-0.69; D of 1.52-2.17 × 10^-3^ mm^2^/s) (56, 150, 153, 182–188). Further, DKI studies have also shown promise in the K value for differentiating breast lesion types, as K was significantly higher in invasive cancers (0.93-0.94) compared to DCIS (0.78-0.81) (56, 188). Nogueira et al. found that K could differentiate a fibroadenoma from fibrocystic change (0.48 vs 0.25) (184).

Histogram analysis has been applied to the kurtosis model in two studies, in which it was found that histogram metrics within each individual group outperformed the mean values, which are typically used in standard diffusion kurtosis imaging (185, 189). Visualization of tumor heterogeneity *via* histogram analysis may result in identification of the most aggressive portions of the lesions and therefore increase diagnostic accuracy in the discrimination of benign and malignant lesions.

In terms of diagnostic performance, few reporting studies demonstrated a high AUC for both D and K in discriminating benign from malignant lesions (153, 182–184). Compared to ADC, kurtosis metrics in some studies demonstrate increased superior diagnostic performance (190), while in others, there was no significant difference (56, 187, 191).

The association of prognostic factors with kurtosis metrics has also been investigated, with studies yielding conflicting results. A few studies found a positive correlation between K and high histologic grade (186, 187, 190), while others showed no association (56, 191). Others also showed significantly increased K value with elevated Ki-67 expression (168, 186, 187, 190), while one found no significant association (108). Studies evaluating the correlation between kurtosis metrics and hormone receptor expression, HER2 status, and lymph node involvement also show varying results (56, 168, 186, 190).

The ability of DKI metrics to predict recurrence risk of breast cancer was evaluated by Wu et. al, and a significant difference was found among multiple histogram kurtosis metrics (D_mean_, D_50%_, K_mean_, K_30%_, K_50%_, K_70%_) and the low, intermediate and high RS groups (192). Specifically, the K_50%_ demonstrated the strongest correlation with risk scores and showed potential as a biomarker for the prediction of breast cancer recurrence.

Overall, the mixed performance of DKI in discriminating lesion malignancy and subtypes warrants critical evaluation into the sources of discrepancies prior to translation into clinical practice. For instance, Mlynarska-Bujny et al. found that residual fat signal from incompletely fat-suppressed DWI images significantly reduced the diagnostic performance of DKI measures and proposed an additional fat correction term to account for fat-related signal contamination (193). Differences in experimental technique (e.g., diffusion time interval), analysis method, ROI selection, and subject variability seem to considerably influence DKI measures. Low SNR from high b-values and long scan times from an increased number of b-values needed for kurtosis modeling have also contributed to fewer clinical studies evaluating DKI (194). Future studies should aim to characterize the variation in DKI across acquisition parameters and provide recommendations for a standardized protocol.

### Synthetic ADC (sADC)

There are several techniques where collecting multiple b-values is desired, however this process consumes scan time. Synthetic or shifted ADC (sADC), potentially addresses the issue of increased scan time by calculating the sADC at two shifted b-values, typically 200 s/mm^2^ and 1500 s/mm^2^, with the aim of capturing both Gaussian and non-Gaussian diffusion (150). A reader study conducted by Iima et al. compared sADC (using b-values=200 and 1500 s/mm^2^) to two integrated diagnostic approaches (combined thresholds approach using IVIM and kurtosis parameters and a Bayesian approach) in the characterization of breast lesions (150). The “combined thresholds” approach calculated the K and ADC at b=0 s/mm^2^ using the kurtosis model and combined them with f_IVIM_ to create a single metric comparable to the BI-RADS score. The Bayesian approach used the f_IVIM_, ADC_0_ and K within each individual lesion to create a probability for BI-RADS categories. The three approaches had high positive predictive value (for radiologists A and B, respectively: combined thresholds, 92.3% and 90.1%; Bayesian approach, 94.6% and 89.7%; and sADC approach, 92.3% and 93.2%), comparable with BI-RADS (93.8%) (150). Furthermore, sADC values differed significantly according to histologic subtypes (P = 0.006). While sADC did not demonstrate higher overall diagnostic performance compared to BI-RADS, the results of the study indicate the parameter’s potential as a non-contrast diagnostic tool. Another study by Choi et al. compared synthetic DWI at b-values of 1000 and 1500 s/mm^2^ with conventional DWI at b-values of 800 and 1500 s/mm^2^ in a group of 50 individuals with breast cancer (195). sDWI_1500_ showed increased lesion conspicuity compared to conventional DWI_1500_, similar to the results of a study by Bickel et al. (196). Although sDWI_1500_ demonstrated decreased overall image quality compared to conventional DWI_1500_, the difference in cancer detection rate was not statistically significant (195). While sADC may demonstrate potential as a rapid alternative to DCE-MRI or conventional DWI, larger studies are needed to better evaluate its diagnostic performance in the breast. The limitation of the synthetic higher b-value is that although it may improve tumor conspicuity, it does not reflect true physiologic assessment associated with real higher b-value data.

### Stretched Exponential Model

**Figure 8:**
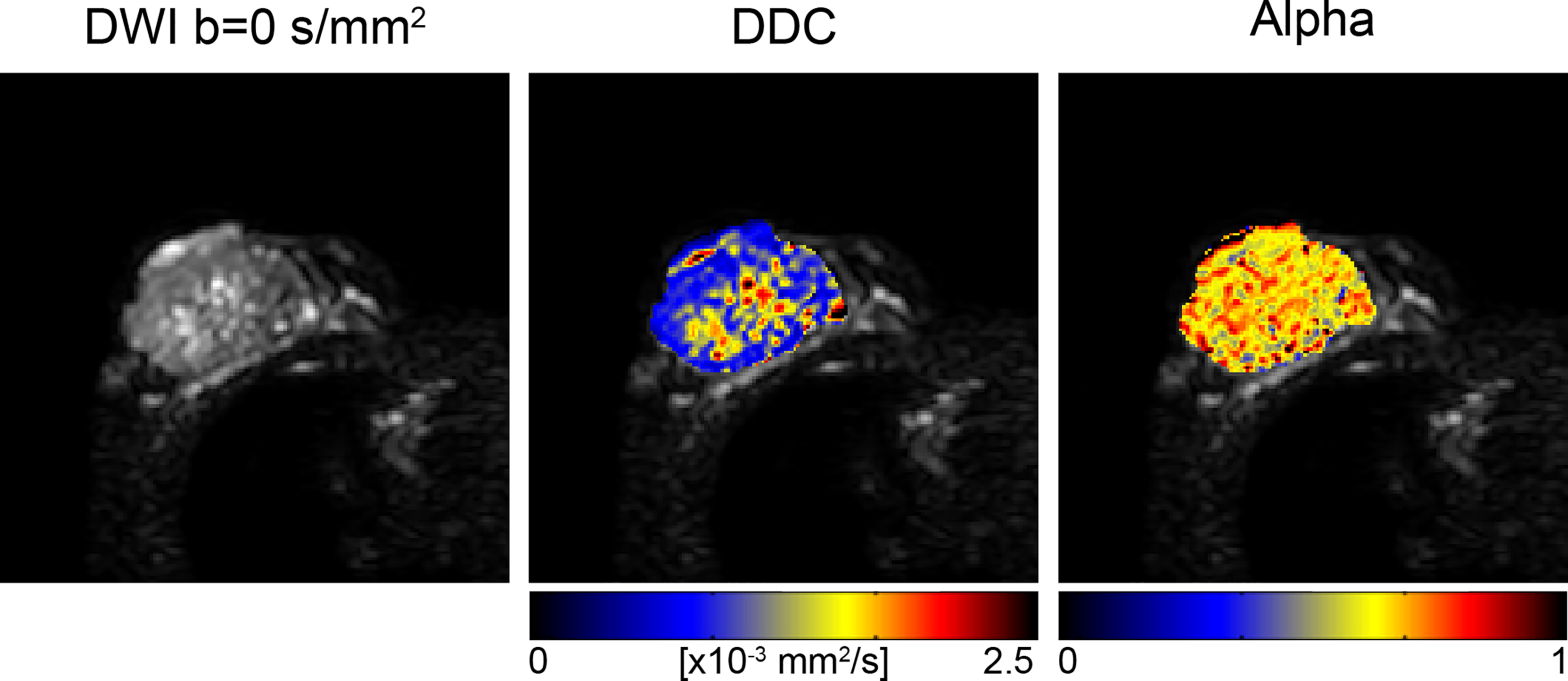
Stretched exponential modeling with b-values of 0, 500, 1000, 1500 and 2000 s/mm^2^ in a 73-year-old patient with invasive ductal carcinoma. Distributed diffusion coefficient (DDC) and alpha maps are overlaid on DWI b=0 s/mm^2^ images, acquired at 3T (56). (Courtesy of Shiteng Suo, PhD, and Jia Hua, MD, Department of Radiology, Ren Ji Hospital, School of Medicine, Shanghai Jiao Tong University, Shanghai, China).

The stretched exponential model is another emerging non-Gaussian diffusion technique that provides added information about diffusion heterogeneity. Parameters include the distributed diffusion coefficient (DDC), which represents the mean intravoxel diffusion rate, and alpha (α), a value between 0 and 1 which quantifies the degree of deviation from monoexponential behavior. An alpha value of 1 represents pure Gaussian diffusion whereas lower values represent diffusion heterogeneity and represent a potential surrogate for tissue complexity (56). Significantly lower DDC and alpha values have been demonstrated in malignant lesions (DDC: 0.72-1.00 × 10^–3^ mm^2^/s, α: 0.62-0.78) compared to benign lesions (DDC: 1.22-1.84 × 10^–3^ mm^2^/s, α: 0.67-0.90) and normal breast tissue (DDC: 1.38-1.83 × 10^–3^ mm^2^/s, α: 0.74-0.86) (**Figure 8**) (56, 197–200). A few studies have also demonstrated that DDC can discriminate invasive breast cancer from DCIS (56, 199) (56).

A study by Suo et al. compared the diagnostic utility of the monoexponential, biexponential, stretched exponential, and kurtosis models in the evaluation of breast lesions (56). The group found a negative correlation between alpha level and tumor size and Ki-67 expression, which is consistent with the hypotheses that larger tumors and those with higher Ki-67 expression (a marker of cellularity) demonstrate increased microperfusion and microstructural heterogeneity. The study also found significantly lower DDC values for ER positive tumors compared to ER negative tumors (0.68 versus 0.77) (56). Regarding goodness-of-fit assessment, the kurtosis model best characterized benign voxels, while the stretched exponential model best characterized malignant voxels. Though multiple non-monoexponential parameters correlated significantly with malignancy, the diagnostic accuracy was not superior to conventional ADC, suggesting that these metrics may provide additional information for tissue characterization but that ADC may remain the standard for breast cancer diagnosis (56).

### Signature Index

Another diffusion weighted technique which may mitigate the issue of complex post-processing and long acquisition times is the Signature index (s-index) proposed by Goto et al., which requires acquisitions at only 3 b-values (201). The S-index is a model free parameter derived from the difference in signal between the tissue in question and a library of reference DW signals for both malignant and benign lesions at two key b-values (201). Using this method, the authors reported comparable diagnostic performance of the S-index and sADC in the discrimination of malignant and benign breast lesions (201). The combination of the S-index with BI-RADS showed the highest diagnostic accuracy. The S-index was also found to correlate with HER2 status and PR expression. One potential drawback is that some of the specificity afforded by individual parameter values that reflect either microvascular or structural changes is lost with the S-index (201).

### Restriction Spectrum Imaging (RSI)

Restriction spectrum imaging (RSI) is an emerging advanced DWI technique that aims to characterize tumor microenvironment based on the behavior of water molecules in different tissue-specific water pools (202–204). The RSI model requires multiple b-values (including b-values up to 4000 s/mm^2^) and diffusion directions at a fixed diffusion time in order to produce maps that differentiate: [1] isotropic restricted (intracellular), [2] anisotropic hindered (extracellular), and [3] free water diffusion compartments (5). This distinction allows for the isolation of diffusion related changes secondary to peritumoral edema or necrosis, which often confounds standard ADC measurements, particularly in the evaluation of aggressive malignancies. In a small group of patients with high grade brain tumors, RSI improved lesion conspicuity and delineation compared to standard DWI (5). Additionally, in the evaluation of tumor response to antiangiogenic treatment in a group of patients with recurrent gliomas, RSI was less affected by medication-induced alterations in edema when compared to ADC, potentially addressing the issue of pseudoresponse and providing a method to identify true tumor response (5).

**Figure 9:**
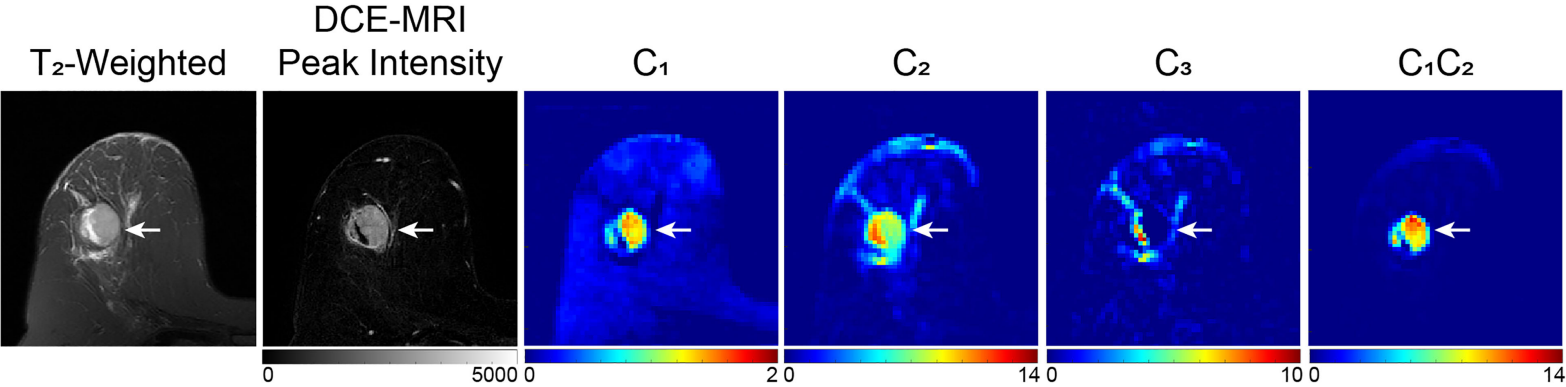
Example of three-compartment RSI analysis in a 49-year-old patient with invasive ductal carcinoma. C_1_, C_2_, and C_3_ maps correspond to the slowest, intermediary, and fastest diffusion compartments, respectively. The lesion, indicated by the white arrow, is hypointense on C_1_ and C_2_ maps compared to surrounding healthy tissue, whereas there is little difference in the C_3_ compartment, which is suggested to correspond to vasculature. The product of C_1_ and C_2_ (C_1_C_2_) results in the greatest tumor conspicuity. DWI images were acquired at b = 0, 500, 1500, and 4000 s/mm^2^ on a 3T scanner, with 50% reduced FOV and without parallel imaging (206).

While initial oncologic applications were in the brain and prostate, the potential role of RSI in breast cancer is actively being explored. Rodríguez-Soto et al. found that a three-component (tri-exponential) RSI model better discriminates malignant lesions from healthy fibroglandular tissue compared to a bi-exponential model and conventional ADC, with similar tumor conspicuity as DCE-MRI (205). In the tri-exponential RSI breast-specific model, the main outputs are signal contribution maps of each compartment C_1_, C_2_ and C_3_. The signal contributions from slow diffusion compartments (C_1_ and C_2_) were larger in malignant lesions than they were in healthy tissue (**Figure 9**) (205). In another study, Andreassen et al. utilized the three-component RSI model to characterize breast lesions in a group of 106 women with pathology-proven breast cancer (206). In this study, the RSI derived parameter C_1_C_2_, representing the product of the signal contributions of the slowest components C_1_ and C_2_, demonstrated comparable diagnostic accuracy to DCE-MRI, with an AUC of 0.984 (206). The false positive rate, given a sensitivity of 80% (FPR_80%_), of the C_1_C_2_ parameter (0.016) was significantly lower than that of conventional ADC (0.731) and K (0.684) (206). It is hypothesized that the higher discriminatory performance of C_1_C_2_ could be attributed to the ability of this parameter to suppress signal from both fibroglandular and fatty tissues, as well as maintain the signal contribution from T_2_ that further differentiates these tissues (206). A case report by Rodríguez-Soto et al. demonstrated the ability of RSI to isolate different water pools in the breast by significantly increasing lesion conspicuity in a lactating woman (high BPE) with biopsy proven IDC compared to both DCE-MRI and conventional DWI (207). Thus, emphasizing the utility of the technique in identifying active disease separate from edema from a lactating breast. Studies of RSI in the breast have thus been performed in patients with known malignancy, and like other diffusion techniques may be challenged in evaluating small lesions. Next steps include adapting RSI to high resolution diffusion imaging, thus allowing the technique to be useful in a screening population (208)

### Time Dependent Diffusion (TDD)

While ADC values obtained from conventional DWI reflect tissue cellularity, it cannot specifically differentiate underlying sub-cellular parameters such as cell size or density (209). Time dependent diffusion, sometimes called temporal diffusion spectroscopy, has shown potential as an emerging parameter to provide added information about the intracellular space, and thereby further characterize tissue biology (209).

Lima et al. demonstrated the time dependence of the ADC value in breast cancer xenografts, with increasing ADC values with increasing diffusion time (210). A few geometric models applied to the diffusion weighted signal, some of which utilize oscillating gradient spin echo (OGSE) acquisitions in addition to pulsed gradient spin echo (PGSE), quantified intracellular diffusion restriction and provided adequate estimates of cell size and intracellular volume (209, 211).

**Figure 10:**
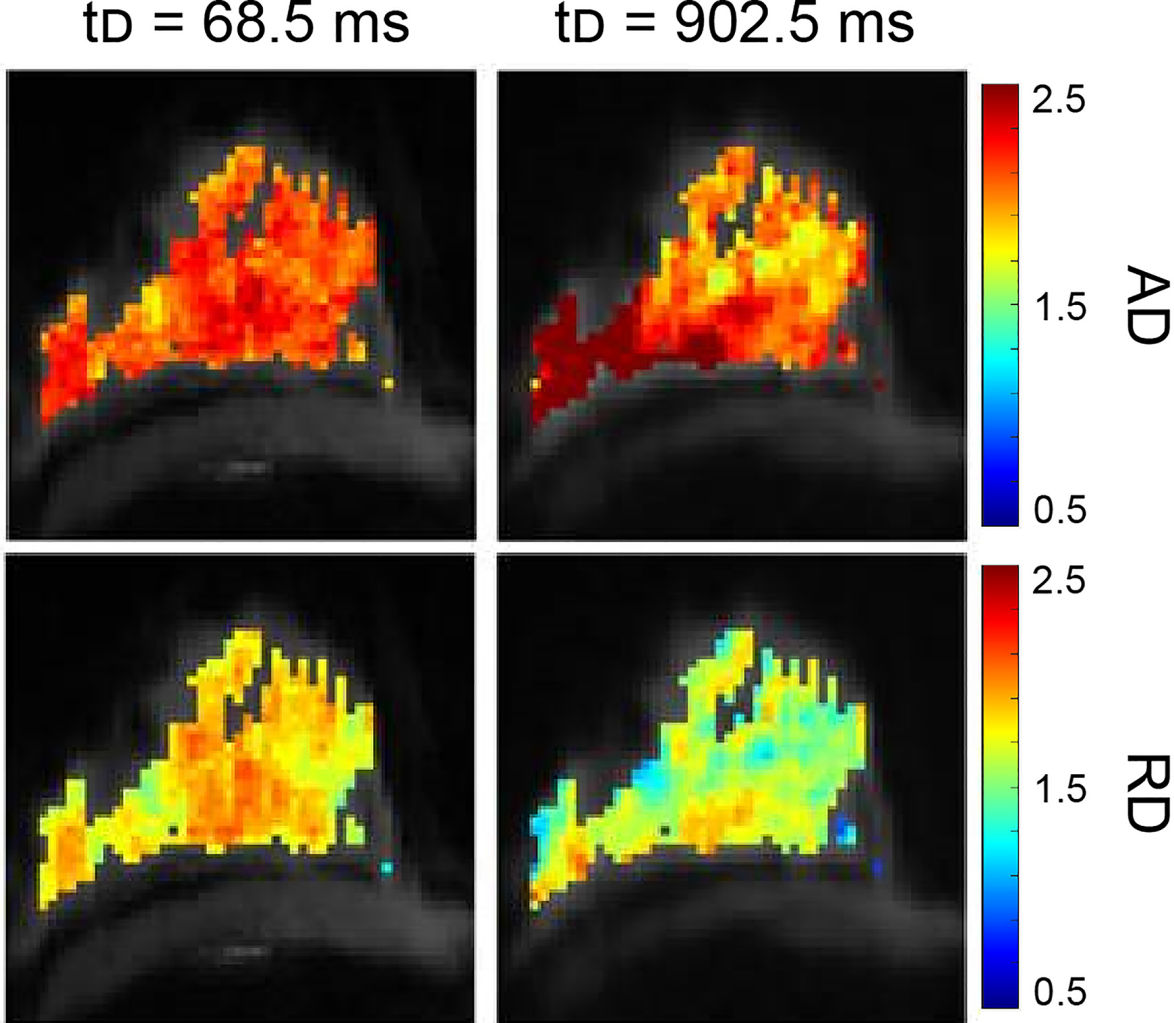
Example of STEAM analysis in a healthy volunteer. The protocol collected a prototype STEAM-DTI sequence with two b-values (0, 500 s/mm^2^) in six directions with parallel imaging in a 3T scanner. Axial diffusivity (AD, first row) and radial diffusivity (RD, second row) [µm^2^/ms] parametric maps at the shortest and longest diffusion time for healthy fibroglandular tissue are shown (212). (Courtesy of Jose Teruel, PhD, Department of Radiation Oncology, NYU Langone Medical Center, New York, New York, USA).

Teruel et al. applied a stimulated echo acquisition mode (STEAM) with multiple diffusion times to normal and pathologic breasts and used a DTI model to fit the data (**Figure 10**). Results showed differences in the estimation of the radial diameter and diffusion length scales for healthy fibroglandular tissue, a simple cyst, and malignant lesions. Complete fat suppression was also seen with longer diffusion times, allowing for more accurate T1 mapping (212).

A few groups have shown that TDD methods increase lesion contrast and may play a role in assessment of treatment response by detecting changes in cell size (211, 213–215). More research is needed to fully understand the application of TDD In breast cancer.

### Radiomics

With the growth of precision medicine comes an opportunity for radiologists to add value by providing relevant information about the patient’s underlying disease in a non-invasive manner. Radiomics is a method of extracting and analyzing large amounts of advanced quantitative data to create a mineable database (216, 217). This data is then used to create analytic and predictive models to correlate radiomic features with diagnostic and prognostic information. Ideally, these radiomic features or signatures would provide insight to the underlying tumor biology and contribute to individualized treatment (216). The standard radiomic process includes 1) image acquisition and reconstruction, 2) image segmentation 3) feature extraction and qualification, and 4) database creation (216, 217).

Ye et al. provided an in depth review of the application of radiomics in breast MRI (216). Although most of the studies were based on DCE modalities, a few were multiparametric and included DWI acquisitions, and even fewer utilized only DWI. For this review, only studies that included DW images will be discussed.

A few groups have evaluated the ability of radiomic models to characterize breast lesions.

Bickelhaupt et al. reported that a Radiomics model based on DKI in the evaluation of mammographic BI-RADS 4 and 5 lesions outperformed ADC and K alone, with improved specificity and a reduction in the number of false positive results by 70% (218). A few multiparametric studies have also demonstrated the ability of radiomic models to differentiate benign from malignant lesions (219–221). Zhang et al. demonstrated an AUC of 0.921 and accuracy of 0.833 in discriminating lesions when using a model based off of T2 weighted, DKI, and quantitative DCE-MRI parameter maps (221). Parekh and Jacobs presented a new radiomic feature mapping framework created from multiple MR sequences and evaluated the utility of this method in the characterization of breast lesions. Authors reported significant differences in textural features between malignant and benign lesions, with an overall sensitivity and specificity of 93% and 85%. Radiomic feature maps provide the added benefit of visual interpretation of feature values as well as lesion heterogeneity (222).

Recent studies have also evaluated the role of radiomics in the prediction of breast cancer subtypes and other prognostic factors. Holli-Helenius et al. reported that the texture features sum entropy and sum variance significantly differed between Luminal A and Luminal B subtypes, with a AUC of 0.876 for the combined radiomic model (223). Other studies have also demonstrated the potential of texture analysis to discriminate among different breast cancer subtypes (224–226). A study by Leithner et al. showed improved accuracy for breast cancer subtype classification when segmentation was performed on the ADC maps, with the highest discriminatory ability seen with Luminal B and HER2 enriched subtypes (227).

In a study by Dong et al., a radiomic model derived from a combination of T2-FS and DWI textural features demonstrated high performance in the prediction of axillary lymph node metastases, with an AUC of 0.863 in the training set and 0.805 in the validation set (228). Another group created predictive models from T1WI, T2WI, DWI and the second post-contrast phase of DCE sequences, and reported an AUC of 0.85 for DWI alone in the prediction of axillary lymph node metastases (229). The highest performance was reported for the model based off of CE2 images with kinetic features, with an AUC of 0.91 (229). No additional performance benefit was found when features from all four sequences were combined, suggesting that DWI radiomic signatures may not play as important a role in the preoperative prediction of axillary lymph node metastases (229).

A few groups have also shown good performance in the ability of radiomic models to predict Ki-67 expression (AUC 0.7 – 0.888) (230–232). A study by Fan et al. found that radiomic analysis of “super resolution” (SR) ADC images better predicted histologic grade and Ki-67 expression compared to features based on conventional ADC images, demonstrating the potential added diagnostic value of a SR technique (233).

The degree to which DWI-based radiomic analyses can predict response to NAC has been investigated by a few groups. Liu et al. found that a radiomics model derived from multiparametric MRI and clinical information better predicted pCR to NAC than individual clinical models and radiomic signatures (234). A model built from pretreatment texture and kinetic parameters significantly helped predict nonresponders with 84% sensitivity in another study (235). A study by Panzeri et al., however, reported no significant correlations between ADC texture radiomic signatures and response to NAC, but parameters derived from DCE-MRI showed utility in predicting response (236).

Comparison across studies is limited as the methods and population sizes used to develop radiomic signatures vary. The role of DWI in radiomics remains under active investigation, many groups demonstrated the potential to aid in the diagnosis, prognosis and surveillance of breast cancer.

### Ultra-High Field Strength

Increasing the field strength is another method in which diffusion-weighted imaging may be improved. The increased CNR and SNR at higher field strengths lead to improved spatial and temporal resolution, which may increase lesion conspicuity and detection. A meta-analysis by Shi et al. included 61 studies comprising 5205 breast lesions and found no significant difference in the diagnostic performance of DWI in the differentiation of malignant and benign lesions at 1.5 T compared to 3 T (32).

**Figure 11:**
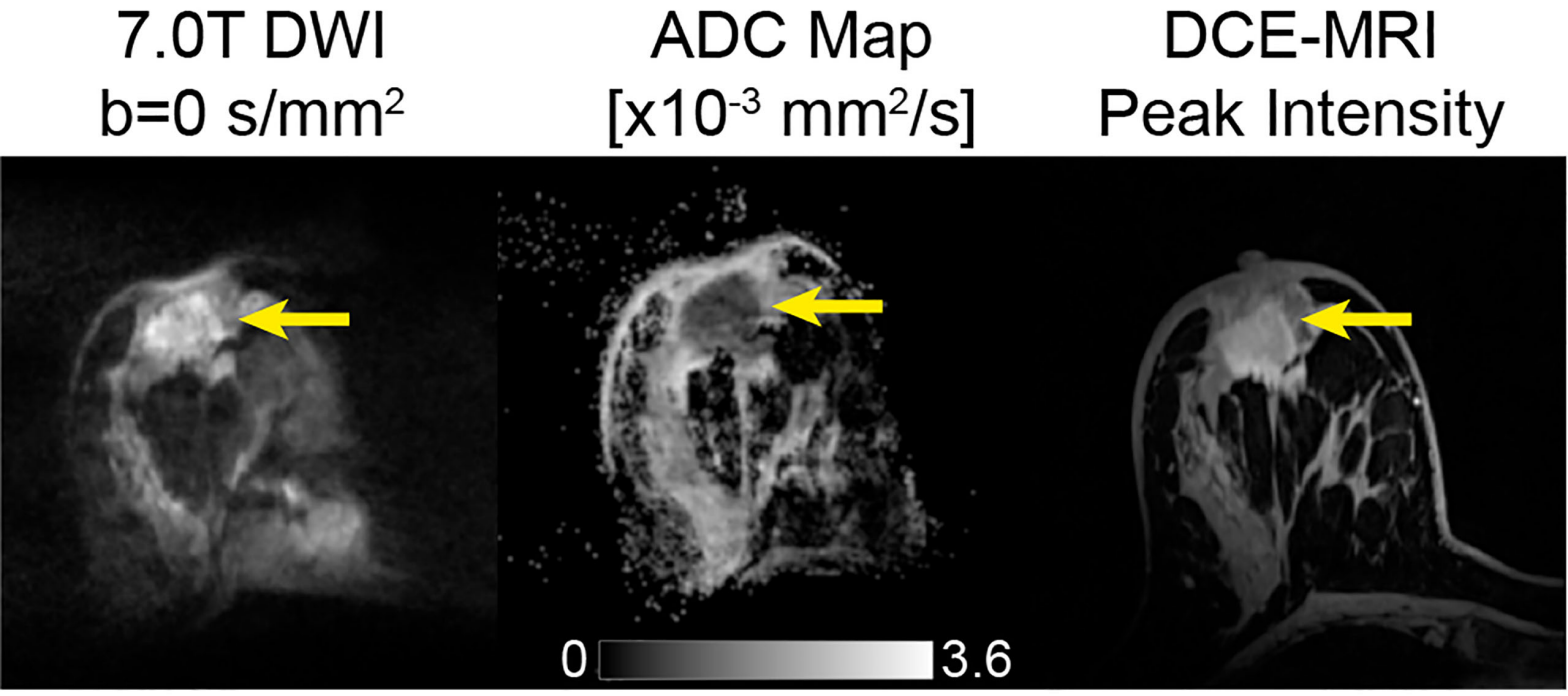
39-year-old female with invasive ductal carcinoma, imaged at 7T using a dedicated four-channel double-tuned ^31^P/^1^H breast coil. Images shown are b=0 s/mm^2^, corresponding ADC map, and DCE-MRI peak intensity subtraction. The mean ADC of the tumor shown is 0.71 ± 0.18 × 10^–3^ mm^2^/s. For DWI, a transverse DWI read-out segmented echo-planar imaging sequence was used. For DCE, patients were intravenously injected with a single dose of contrast agent (gadoterate meglumine) as a bolus, followed by a 20-mL saline flush after three of the 18 repetitions of the time-resolved angiographic imaging with stochastic trajectories sequence (26). (Courtesy of Katja Pinker-Domenig, MD, PhD, Department of Radiology, Memorial Sloan Kettering Cancer Center, New York, New York, USA).

Several technical limitations arise when increasing the field strength, particularly at ultra-high fields (7T and above). At 7T, DWI must overcome limitations due to the increased specific absorption rate (SAR) in addition to heterogeneous fat suppression and T2* blurring, which degrade image quality. A few groups have mitigated these issues through the use of bilateral coil designs and demonstrated the feasibility of DWI of the breast at 7T (**Figure 11**) (26, 237–240).

Bogner et al. showed that combination of rs-EPI DWI with parallel imaging at 7T significantly reduced artifacts and improved image quality, with submillimeter resolution and good diagnostic performance in the characterization of breast lesions (238). A study by Gruber et al. compared DWI of breast lesions at 7T versus 3T using rs-EPI and found increased sensitivity (100% compared to 94%) at 7T for the same ADC threshold and specificity, comparable SNR and CNR, and a 2.4 times higher spatial resolution. These findings suggest that 7T may aid in the detection of smaller lesions that otherwise are more difficult to visualize (239).

Multiparametric MRI of the breast at 7T was also performed by a few groups (26, 237, 240). Of note, Pinker et al. generated excellent quality images and found that multiparametric MR eliminated false negative findings and decreased the number of false positive findings in 40 women (26). Clinically, this may translate into a reduced number of unnecessary biopsies and improved diagnostic accuracy.

Though most of the literature consists of a few studies with small sample sizes, the results are promising with the main limitation being the general lack of accessibility to 7T scanners for most patients (and breast imaging researchers).

## Discussion

An abundance of evidence has shown the utility of DWI as an imaging biomarker for breast cancer, with applications ranging from screening, lesion detection and characterization, and treatment response evaluation. The monoexponential ADC has shown promise in differentiating benign and malignant lesions; however, significant overlap in reported ADC ranges for these tissues limit the clinical utility of ADC cutoffs. Further, there have been conflicting results in the ability of ADC in discriminating lesion subtypes, likely owing to varying study design and protocol differences. Recently, the ACRIN 6698 trial showed high precision in a multi-institution and multi-platform setting, marking a milestone in the validation of DWI as a biomarker in breast imaging and highlighting the need for standardized protocols. As a result, a breast section was incorporated into the 2019 QIBA profile, providing guidance for implementation in community practice.

As described throughout this review, some advanced DWI models require data acquisition at high b-values, which increases susceptibility to B_0_ inhomogeneity-induced artifacts and noise, especially for EPI-based sequences. Thus, several methods have been proposed to address this issue, but are beyond the scope of this review (241). An additional limitation of breast DWI is that due to the breast’s underlying tissue complexity (e.g. intricate composition of fat, fibroglandular tissue and cancers), measurements of DWI-derived parameters in tissues different from fat tend to be underestimated unless adequate fat suppression is achieved (182). Moreover, the monoexponential ADC, which assumes Gaussian diffusion, may not completely capture the complex diffusivity properties of the breast, especially in lesions which display increased tissue heterogeneity. This may explain the conflicting results observed across multiple studies.

To circumvent the limitations of standard ADC, advanced diffusion modeling techniques such as DKI, DTI, IVIM, and RSI may provide added information on the underlying microenvironment by characterizing the non-Gaussian diffusion within tissues. Although promising, the advanced modeling techniques discussed in this paper require further validation through multi-institution studies, optimization of protocol parameters, and demonstration of repeatability and reproducibility prior to use in clinical practice.

With continued research on methods to improve standard DWI, such as increasing field strength and alternative acquisition techniques, advanced modelling techniques, and radiomics, DWI may play an increasingly important role in the evaluation of breast cancer.

## Author Contributions

AM: this author was responsible for interpreting the relevant literature and drafting the majority of the review. LF: this author was responsible for revising portions of the review and creating the figures. CM: this author was responsible for drafting and revising portions of the review. SB: this author was responsible for revising portions of the review. SL: this author was responsible for generating data and creating figures. AR-S: this author was responsible for critically revising the review and serving as an expert in the field. RR-P: this author was responsible for critically revising the review, serving as an expert in the field, and approving the final draft. All authors contributed to the article and approved the submitted version.

## Funding

This work was supported by the California Breast Cancer Research Program [24IB-0056 IDEA Award] and the Krueger v. Wyeth Research Award, and the National Cancer Institute R37 CA249659, and GE Research. The funder was not involved in the study design, collection, analysis, interpretation of data, the writing of this article or the decision to submit it for publication.

## Conflict of Interest

RR-P: Human Longevity Inc: Consultant, Cortech Labs: Stock options, Curemetrix: Stock options, consultant. and GE: research agreement.

The remaining authors declare that the research was conducted in the absence of any commercial or financial relationships that could be construed as a potential conflict of interest.

## Publisher’s Note

All claims expressed in this article are solely those of the authors and do not necessarily represent those of their affiliated organizations, or those of the publisher, the editors and the reviewers. Any product that may be evaluated in this article, or claim that may be made by its manufacturer, is not guaranteed or endorsed by the publisher.
